# Fold-Over Oversampling Effects in the Measurements of Cerebral Cerebrospinal Fluid and Blood Flows with 2D Cine Phase-Contrast MRI

**DOI:** 10.3390/diagnostics10060387

**Published:** 2020-06-09

**Authors:** Fadoua Saadani-Makki, Serge Metanbou, Garance Arbeaumont-Trocme, Julien Van Gysel, Malek I. Makki

**Affiliations:** 1 MRI Research GIE-FF, CHU Amiens Picardie, 80054 Amiens, France; makki.fadoua@chu-amiens.fr (F.S.-M.); manipfairefaces@chu-amiens.fr (G.A.-T.); vangysel.julien@chu-amiens.fr (J.V.G.); 2 CHIMERE EA7516, University Picardy Jules Verne, 80025 Amiens, France; 3 Pole Imagerie, CHU Amiens Picardie, 80054 Amiens, France; metanbu.serge@chu-amiens.fr

**Keywords:** cine-phase contrast, phase-offset, fold-over oversampling, brain dynamics

## Abstract

This prospective study investigated the effects of fold-over oversampling on phase-offset background errors with 2D-Cine phase contrast (Cine-PC) magnetic resonance imaging (MRI). It was performed on brain MRI and compared to conventional Full-field of view FOV coverage and it was tested with two different velocity encoding (Venc) values. We chose Venc = 100 mm/s to encode cerebrospinal fluid (CSF) flows in the aqueduct and 600 mm/s to encode blood flow in the carotid artery. Cine-PC was carried out on 10 healthy adult volunteers followed simultaneously by an acquisition on static agar-gel phantom to measure the phase-offset background errors. Pixel-wise correction of both the CSF and the blood flows was calculated through 32 points of the cardiac-cycle. We compared the velocity-to-noise ratio, the section area, the absolute and the corrected velocity (peak; mean and minimum), the net flow, and the stroke volume before and after correction. We performed the statistical T-test to compare Full-FOV and fold-over and Bland–Altman plots to analyze their differences. Our results showed that following phase-offset error correction, the blood stroke-volume was significantly higher with Full-FOV compared to fold-over. We observed a significantly higher CSF mean velocity and net flow values in the fold-over option. Compared to Full-FOV, fold-over provides a significantly larger section area and significantly lower peak velocity-offset in the aqueduct. No significant difference between the two coverages was reported before and after phase-offset in blood flow measurements. In conclusion, fold-over oversampling can be chosen as an alternative to increase spatial resolution and accurate cerebral flow quantification in Cine-PC.

## 1. Introduction

Flow-encoding 2D-Cine phase-contrast (Cine-PC) magnetic resonance imaging (MRI) has growing interest in cerebrospinal fluid (CSF) and blood flow assessment. It has the capability to measure the peak and mean flow velocities and volumes through the entire cardiac cycle. These underlying flow information and dynamic parameters can be used to grade the severity of hydrocephalus pathologies and cerebrovascular artero-venous dysfunctions. CSF and blood flow quantifications have been successfully performed in the aqueduct [1] and the artero-venous systems [2]. These measurements allow for a better understanding of the brain hydro/hemo dynamic related to hydrocephalus intracranial hypo/hypertension [3] and posterior fossa cystic mal-formations [4]. Recent Cine-PC study on healthy controls has demonstrated a regression of blood flow and velocity with age [2]. It has been successfully applied to reproduce the distribution of blood flow in the branches of the circle of Willis with age [5]. The caveat of Cine-PC is that it applies bipolar flow-encoding gradients that generate phase-offset errors due to eddy-currents (rapid switching from positive maximum to negative maximum), concomitant magnetic field gradients (Maxwell terms), and gradient field distortions (non-linear gradients) [6]. When combined together and integrated over time, these inherent background errors produced inaccuracy in the assessment of flow measurements and characteristics and were more prominent in high-power gradient systems [7,8]. Optimal acquisition sequences involving corrections for Maxwell terms, gradient amplifier, and eddy-current induced velocity-offset are essential for accurate flow parameter quantification in small intracranial vessels or in the aqueduct [9]. An acquisition of Cine-PC images on static phantom might be the ultimate solution to reduce these remnants of phase-offset errors. This can be achieved through direct pixel-wise subtraction of the measured “static” velocity from the volunteers’ measures to compensate for induced velocity errors [7]. While substantial numbers of Cine-PC studies have been applied in cerebrovascular diseases, no investigation has estimated the phase-offset errors in small intracerebral vessels (arteries, veins and aqueduct) with low velocity-encoding (*Venc)* values (<1000 mm/s). This is crucial because of the higher demand on flow-encoding gradient capabilities to obtain greater velocity-to-noise ratio (VNR). Furthermore, to achieve a high standard of accuracy and precision in these measurements, a better extraction of the section area is required to minimize the partial volume effects. This requires the acquisition of a high spatial resolution image. A reduced field-of-view (FOV) prescription is an option to increase the spatial resolution, but it might generate wrap-around artifacts and phase discontinuity in the parametric flow maps. The results of such artifacts are large fitting errors that amplify the phase-offset errors around the region of interest. 

The wrap-around artifact arises when the prescribed image acquisition FOV covers part of the anatomy to be scanned instead of the whole object. The result is a reproduction of the tissues outside the FOV at the opposite edge of the scan. This artifact (i.e., aliasing) occurs mainly in the phase-encode direction and it generates when the sampling frequency violates the Nyquist law (sampling rate must be at least twice the maximum signal frequency). The “fold-over” suppression option [8], also known as no-phase-wrap, is an anti-aliasing technique that eliminates fold-over artifacts in the magnitude images by increasing the distance over which objects may fold back on both sides of the small FOV while maintaining the same bandwidth (BW) values. On one hand, this requires doubling the number of phase-encoding matrix size, and on the other hand, one has to halve the number of excitation so that the acquisition time and the signal-to-noise ratio (SNR) remain unchanged. 

The purpose of this study was to investigate the potential advantage of the “fold-over” oversampling option (i.e., no phase-wrap) compared to Full-FOV coverage in the acquisition of Cine-PC MRI. To achieve this aim, first, we measured the phase-offset errors in both coverage methods with two different velocity encoding values using a static gel phantom, and second, we compared the dynamic of acqueductal CSF and arterial blood of the two coverage modes in heathy subjects before and after phase-offset correction.

## 2. Materials and Methods

### 2.1. Theory

The signal intensity of the effective magnetization that satisfies the Nyquist theorem equals the complex sum of the magnetization of all spins [10]. For *Y* phase encoding lines that are recorded sequentially, the signal intensity *S*, at certain step *m*, reads

(1) $$S\left(m\right)=\overset{Y-1}{\underset{y=0}{\sum }}M_{T}\left(y.∆k\right).exp\left(\frac{-2\pi iym}{Y}\right).exp\left(\frac{-\pi iy\left(Y-1\right)}{NY}\right)$$

where *M_T_* is the magnetization in the transverse plane

Δ*k* is the pixel size, and *m = 0, 1, …, Y − 1*

**Figure 1:**
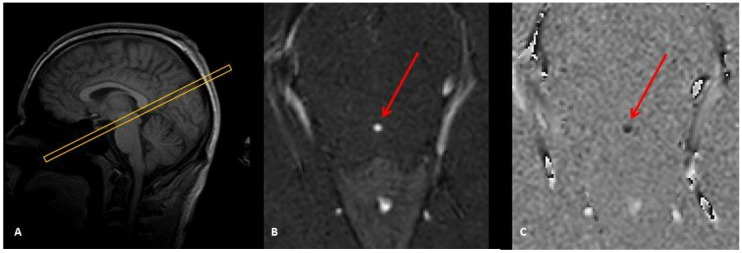
Sagittal image scout (**A**) to localize the aqueduct and to prescribe a perpendicular 2D Cine-phase MRI slice. This provides a magnitude image (**B**) suited to visualize and select the aqueduct (red arrow) and a phase-difference image to map the CSF flow (**C**). (B) and (C) represent one phase of the cardiac cycle.

For magnitude image reconstruction (Figure 1), the term *φ(y) = exp[-i.π.y.(Y − 1)/Y]* is simply removed because it is part of the signal phase and does not contribute to the amplitude of the signal. Thus, after *m* steps, the magnitude images are reconstructed with the amplitude of the modified Equation (1) that reads

(2) $$S\left(m\right)=\overset{N-1}{\underset{y=0}{\sum }}M_{T}\left(y.∆k\right).exp\left(\frac{-2\pi iym}{Y}\right)$$

Flow mapping images or phase-differences (Figure 1C) result from the subtraction of two datasets. The first dataset is acquired with bipolar phase-encoding gradients in a specific direction (inferior–superior, left–right, or anterior–posterior) and the second is obtained by toggling these bipolar gradients to acquire the same image in the opposite direction (respectively, superior–inferior, right–left, posterior–anterior). The axis of the bipolar direction is user defined, and it can be chosen in any of the three physical axes or all combined together. In this phase-difference technique, the term *φ(n)* cannot be merely neglected and must be accounted for. In fact, the unpredictable behavioral of phase-shift in each acquired image might affect the flow mapping dataset. 

### 2.2. Image Acquisition

The study was performed on a 3T Achieva dStream scanner (Philips Healthcare, Best, The Netherlands) equipped with a gradient strength *G_0_* = 40 mT/m and a slew-rate *SR* = 200 T/m/s. The imaging parameters were: Cine-PC fast-field-echo (FFE) sequence, Cartesian filling with flow-compensation, Sense = 1.5, flip angle = 30°, and a 2 mm slice thickness. A comparative list of the imaging parameters between the two schemes is given in Table 1. An axial oblique slice was prescribed perpendicular to the aqueduct with a through plane *Venc* value of 100 mm/s to encode CSF flow velocity (Figure 1). For blood flow analysis, we chose the internal carotid artery (ICA) and *Venc* = 600 mm/s. The slice was prescribed perpendicular to the ICA at the level of the cervical spine C2–C3. Choosing either the jugular vein or the vertebral artery would answer the questions raised by this investigation. Since the main purpose of this study was to mimic the phase-offset errors on blood flow, there was no need to assess the total cerebral blood flow. The coverage-based comparison involved: (1) a full coverage of the whole head, referred as Full-FOV, and (2) a small FOV referred to as fold-over oversampling to remedy for aliasing. The experiments were performed on ten healthy volunteers with informed consent. The group included ten controls (25–55 years of age, five females) recruited in our university hospital. The inclusion criteria were no history of any neurologic disorder, or neurological development, or head trauma, and could hold still in the scanner. For the purpose of our study, a static phantom was filled with 3 L of water solidified with commercial 16 g of agar powder and 1 kg of sugar. Simultaneously after each acquisition from the volunteers, the phantom was scanned using the same imaging parameters and slice positions to measure the static phase-offset errors.

### 2.3. Image Processing and Analysis

We analyzed the VNR, the mean, the peak, and the minimum velocity-offset measured on the static phantom (V_peak_^offset^, V_mean_^offset^, V_min_^offset^). On each of the ten volunteers, we quantified the mean, peak, and minimum absolute (i.e., uncorrected) velocities (V_peak_, V_mean_, V_min_). We extracted the aqueduct and the ICA section areas (mm^2^), the absolute net flows (Q_Net_), and the stroke volume (SVol). The final step aimed to correct the variables measured on the volunteers through baseline subtraction of the phase-offset errors measured on the static phantom. We calculated V_peak_^corr^, V_mean_^corr^, V_min_^corr^ Q_Net_^corr^, and SVol^corr^ as follows:

(3) $$X_{R}^{corr}=X_{R}^{}-X_{R}^{offset}$$

where *R* stands for peak, mean, min, or Net; *X* stands for Velocity (V), net flow (Q), or stroke volume SVol.

These flow measures were processed with a semi-automatic delineation of the section areas through the entire cardiac cycle.

The paired 2-tailed T-test statistical test was carried out to analyze the differences between Full-FOV and Fold-Over for each dynamic variable. The Pearson bivariate test (SPSS v22, IBM Chicago, IL) was performed to assess the correlations of these variables measured with the two coverage options. Bland–Altman plots were generated to test the lower and upper limit of differences and agreements between Full-FOV and fold-over. These statistical analyses were performed for the 2 prescribed *Venc*.

## 3. Results

**Figure 2:**
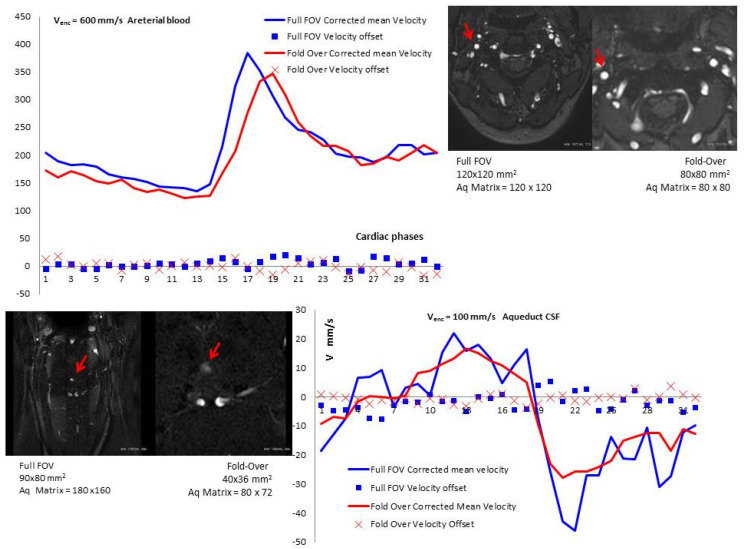
The top graph shows the curves of mean blood flow velocity in the internal carotid artery (*Venc* = 600 mm/s) after subtraction of the velocity offset (*X*-axis represents the 32 cardiac phases). The lower graph shows the curve pattern of mean aqueductal CSF flow velocity (*Venc* = 100 mm/s) through the 32 cardiac phases after subtraction of the velocity offset recorded in the phantom for both coverage modes (Full FOV and fold-over). These two graphs are the results of the same volunteer.

Similar patterns of corrected mean velocity values (V_mean_^corr^) were observed in both coverage modes (Full-FOV and fold-over) with the two *Venc* values (Figure 2). Following phase-offset error correction, there were significant differences in the CSF flow mean velocity (V_mean_, *p =* 0.009) and the CSF net flow volume (Q_Net_, *p =* 0.02) with a fold-over option. These differences were not significant with Full-FOV coverage. No difference was recorded in the ICA blood flow (Table 2). The main significant results and most important findings are detailed in Table 2.

### 3.1. Section Area

The ICA section area was not significantly different between the two coverage options (Full-FOV vs. fold-over). The aqueduct section area was significantly higher (*p <* 0.01) using fold-over (3.74 ± 1.30 mm^2^) compared to Full-FOV (2.86 ± 0.98 mm^2^) (Table 3). There were strong correlations between the two coverage options in the ICA section areas (*r* = 0.80, *p =* 0.006) and the aqueduct section area (*r* = 0.91, *p <* 0.001) (Table 4).

### 3.2. Velocity-to-Noise Ratio (VNR)

Higher, but non-significant VNR was measured in fold-over compared to Full-FOV with the two *Venc* values. The VNR measured in the aqueduct with fold-over correlates significantly with that of Full-FOV (*r* = 0.68, *p =* 0.03) (Table 4).

### 3.3. V_peak_, V_peak_^offset^, and V_peak_^corr^

**Figure 3:**
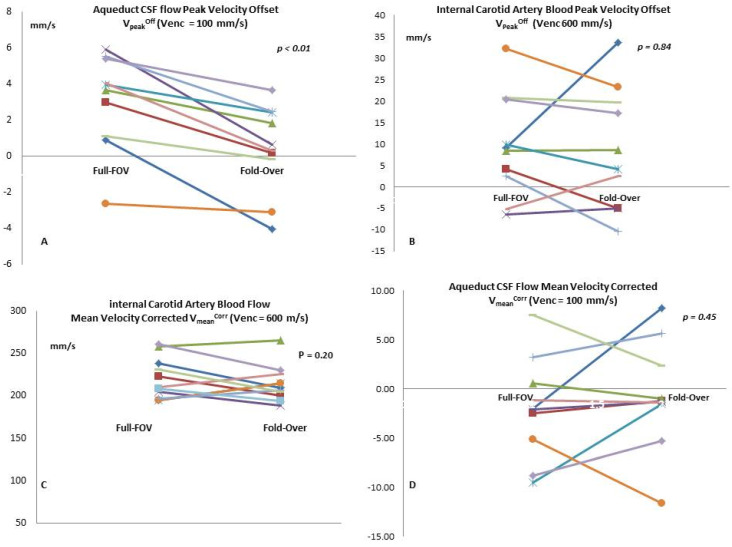
These comparative plots represent the measures for each volunteer. The graphs demonstrate the differences between Full-FOV and fold-over when we measured the peak velocity offset in the aqueductal CSF (**A**) and the internal carotid artery blood (**B**). We also show the difference in flow mean velocity following phase-offset correction in the aqueduct CSF (**C**) and internal carotid arterial blood (**D**).

Compared to Full-FOV, the fold-over option provided significantly lower CSF peak velocity offset (V_peak_^offset^) *(p =* 0.03) (Figure 3). The absolute peak velocity (V_peak_) measured in the aqueduct and in the ICA did not differ statistically (Table 3). Similarly, we did not observe any significant difference in the corrected peak velocity (V_peak_^corr^), neither in the CSF nor in the blood flow (Table 3). V_peak_^offset^ measured with fold-over correlated significantly (Table 3) with that of Full-FOV in the ICA blood flow (*r = 0.68, p =* 0.030) and in the aqueduct CSF flow (*r =* 0.81, *p =* 0.005). A strong correlation between the two coverage options was observed in the V_peak_ of the blood flow (*r* = 0.66, *p* = 0.039) (Table 3) and V_peak_^Corr^ in the CSF flow (*r =* 0.90, *p <* 0.001).

### 3.4. V_mean_, V_mean_^offset^, and V_mean_^corr^

Lower, but non-significant mean velocity offset (V_mean_^offset^) value was measured with the fold-over option and the two *Venc* values. The volunteers’ mean velocity (V_mean_) did not statistically differ in the CSF flow or blood flow. Following phase-offset correction, we did not observe any significant difference in the corrected mean velocity (V_mean_^corr^) in the CSF or in blood flow (Table 3). There were significant correlations (Table 3) between the two coverage options in the V_mean_^offset^ of ICA blood (*r =* 0.84, *p =* 0.002) and aqueduct CSF (*r =* 0.75, *p =* 0.012).

### 3.5. V_min_, V_min_^offset^, and V_min_^corr^

Lower, but non-significant minimum velocity offset (V_min_^offset^) was measured with fold-over option using the two *Venc* values. The volunteers’ minimum velocity (V_min_) did not statistically differ in CSF flow or in blood flow. Following phase-offset correction, we did not observe any significant difference in the corrected minimum velocity (V_min_^corr^) in CSF flow or in blood flow (Table 3). There were significant correlations between the two coverage options (Table 4) in V_min_^offset^ in both ICA blood flow (*r =* 0.88, *p <* 0.001) and aqueduct CSF flow (*r =* 0.64, *p =* 0.045). As a result, the blood flow V_min_^corr^ and V_min_ measured with fold-over correlated significantly with that of Full-FOV (*r =* 0.80, *p =* 0.005; and *r =* 0.82 *p =* 0.003).

### 3.6. Q_Net_ and Q_Net_^corr^

The comparison between the two coverage modes did not record any significant difference in the net flow volume (Q_Net_) in CSF or in blood (Table 4). The same observations were reported following phase-offset correction Q_Net_^corr^. The blood flow Q_Net_ measured with the two coverage options correlated significantly (*r* = 0.85, *p =* 0.002) unlike the Q_Net_^corr^. No correlation was reported in Q_Net_^corr^ or Q_Net_ of the aqueduct CSF flow (Table 4).

### 3.7. SVol, and SVol^corr^

In the aqueduct CSF flow, the comparison between Full-FOV and fold-over demonstrated that there was no significant difference in the stroke volume before or after phase-offset correction (SVol, SVol^corr^). The ICA blood SVol and SVol^corr^ were not significantly different when comparing the two coverage options. Significant correlations between Full-FOV and fold-over were observed in the ICA blood flow SVol and SVol^corr^ (Table 4).

### 3.8. Bland–Altman

**Figure 4:**
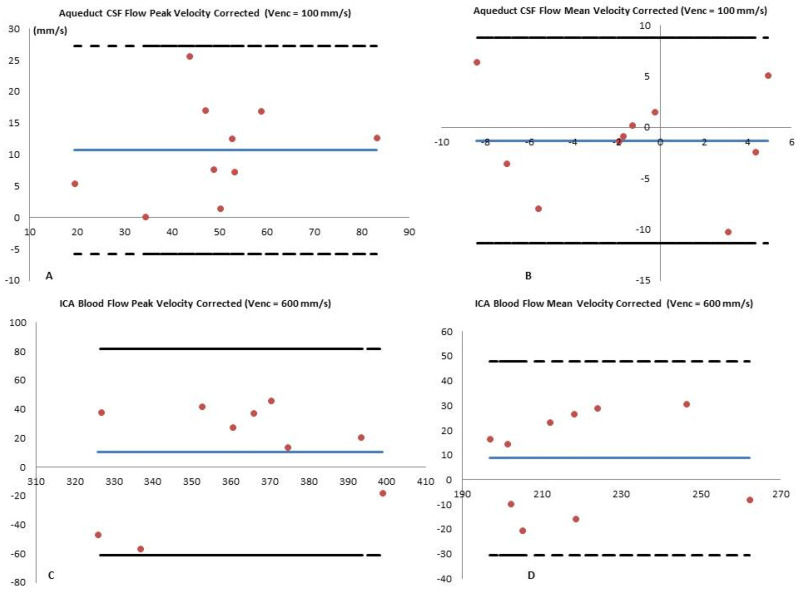
Bland–Altman analysis of Full-FOV versus fold-over performed in the corrected mean CSF flow velocity V_mean_^corr^ measured at the level of the aqueduct (**A**) and blood flow in the internal carotid artery (**B**). Similar analysis results are shown for stroke volume corrected values SVol^corr^ in the CSF (**C**) and in the blood flows (**D**). The plain blue lines represent the mean values measured by the two coverage options, and the red dots represent the mean difference between the two coverages [Full-FOV—fold-over]. The dashed lines correspond to the higher limit (mean + 1.96 × standard deviation) and the lower limit (mean − 1.96 × standard deviation). The X-axes refer to the mean values of the two measures and the Y-axes refer to the mean differences between the two measures.

Bland–Altman analysis [11] showed a small systemic difference between Full-FOV and fold-over when measuring V_mean_^corr^ and V_peak_^corr^ (Figure 4). In the ICA blood flow, the bias was under 1% for all variables except for the net flow Q_Net_ (2.11%, 2.05 μL.s^−1^) and the net flow corrected Q_Net_^Corr^ (3.78% 4.83 μL.s^−1^). In the aqueduct CSF flow, the bias fluctuation was more pronounced (Table 4) and it reached 71% in the V_min_ (11.03 mm.s^−1^) and 3.99% in V_min_^Corr^ (−0.59 mm.s^−1^). We measured a −13.35% bias in the V_mean_^Off^ (0.17 mm.s^−1^) and a 6.68% in the V_mean_ (2.27 mm.s^−1^). The peak velocity biases were as follows: V_peak_^Off^ (1.83 %, 0.05 mm.s^−1^), V_peak_ (4.3%, 0.63 mm.s^−1^), and V_peak_^Corr^ (2.42%, 0.26 mm.s^−1^).

## 4. Discussion

This study aimed to investigate the effects of fold-over oversampling compared to Full-FOV coverage in the assessment of CSF and blood flow dynamics with Cine-PC MRI. We demonstrated that the VNR, the mean velocities, the net flows, and the stroke volumes were not significantly different. This was observed with two different *Venc* values (100 and 600 mm/s) prescribed respectively at the level of the aqueduct and the internal carotid artery. Following phase-offset corrections, we recorded significantly higher blood flow stroke volume with Full-FOV mode. In the CSF flow, we noticed that following phase-offset correction, the net flow volume and the mean flow velocity were significantly higher. The fold-over option provides a significantly lower V_peak_^offset^ in the aqueduct CSF flow. However, V_peak_ and V_peak_^corr^ were not significantly different. Given the important contribution of concomitant gradient fields and eddy-current induced offsets to overall phase errors, particularly with low *Venc* and oversampling coverage, there is a need to explicitly subtract these from Cine-PC data. In cardiovascular MRI, phantom corrections of blood flow measurements often resulted in clinically significant changes. Following phase-offset correction, the flow measurements in patients with known or suspected congenital heart disease have shown that up to 12% of Fallot patients have been reclassified according to the severity of the pulmonary regurgitation [12]. Another study pointed out that 13% to 48% of flow measurements were sufficiently affected by phantom correction, enough to potentially alter clinical management [13]. Intracranial blood and CSF flow velocities were lower than those of cardiovascular systems and they require reduced velocity sensitivity compared to that prescribed in CMR (>2000 mm/s), hence stronger flow encoding gradients are needed. This involves more contribution of eddy-currents and Maxwell-effects in the inaccuracy and the oscillation of the measured values. It has been shown that velocities measured with lower *Venc* are more susceptible to errors from intra-voxel dephasing and are of concern when quantifying CSF or cerebrovascular flows [14,15,16]. The lower values reported by non-corrected measures resulted from an increased noise, which led to erroneous velocity measurements, particularly in regions of slow flow. This is in line with previous studies showing that an automatic phase unwrapping often failed to correct for phase errors with a low *Venc* [13,17]. Compared to Full-FOV, fold-over oversampling significantly increased CSF V_peak_^offset^. The impact of velocity-offset correction led to higher CSF and blood net flow values (Q_Net_^corr^ > Q_Net_). This highlights the importance of such correction to better rate patients with altered cerebral hydrodynamic and/or hemodynamic. Recent studies on idiopathic normal pressure hydrocephalus have shown that there was a reversed Q_Net_ direction compared to the healthy controls [18], or patient following surgical shunt [19]. Other studies have shown no difference in terms of Q_Net_ magnitude and directions [20]. An error of approximately ±10% in Q_Net_ in aqueductal flow rate was reported as acceptable [21], nevertheless a decrease of such errors is mandatory. The outcome is a better evaluation of complex cerebrovascular diseases such as arteriovenous malformations and alteration of the CSF hydrodynamic seen in hydrocephalus. Overall, the measurements of blood and CSF Q_Net_ showed a good correlation between Full-FOV and fold-over and the values were within acceptable limits of agreements.

Many errors influence the quantification of Cine-PC parameters. These could be generated by any of the following: VNR, inflow effects, changes in the pulsatility, physiological factors, and partial volume effects. A caveat of these quantifications is that inter-variability might be larger than intra-variability in both patients and healthy control subjects [22]. Higher spatio-temporal resolution is critical for such applications to better delineate the ROI and to enhance the accuracy of these measurements over the entire cardiac cycle [19,20]. The trade-off is an increase of eddy-currents effects and gradient non-linearities that result from gradient field inhomogeneity. These small systematic inaccuracies seen in individual cardiac phase might be of concern when they propagate to the entire cardiac cycle. The fold-over option might be a compromise between higher spatial resolution and phase-offset errors. When Cine-PC is performed to measure CSF flow in the aqueduct (diameter ~2 mm), the fold-over oversampling option provides significantly a larger section area compared to Full-FOV. A better delineation of the aqueduct section area achieves accurate CSF measurements through a reduction in the partial volume effects, while inaccurate delineation of the aqueductal section area might lead to ±23.1% variations in Q_Net_ using high spatial resolution [23]. For cerebrovascular blood flow assessment, the fold-over option is similar to Full-FOV with regard to the section area and does not impact the delineation.

VNR is inversely proportional to the magnitude of *Venc,* hence when Cine-PC is performed to measure flow with a low velocity dynamic range (cerebrovascular blood or CSF), the prescribed *Venc* produces relatively high VNR in the targeted flow region [24]. In this investigation, the two different coverages demonstrated that there was no statistical difference in VNR, thus fold-over should not be selected solely for the purpose of increasing VNR value.

The higher CSF SVol^corr^ recorded with fold-over in the aqueduct resulted from an increase in both the section area and the V_mean_^corr^. Previous study by Yoshida et al. (2009) pointed out that low spatial resolution overestimated SVol due to partial volume effects and larger section area [21]. Other investigators stipulated that aqueductal SVol was more sensitive to inaccuracies from the calculation of V_mean_, rather than manual delineation of the section area [19]. In our study, we showed that the fold-over option provided a larger (respectively smaller) section area, and SVol was higher (respectively lower).

Our results demonstrated that fold-over overcomes wrap-around artifact and decreases inaccuracy [23], thus it can be activated with the need for background phase-offset compensation. The trade-off is a slight increase in both TE and TR. The difference reported between fold-over and Full-FOV is mainly related to the acquisition mode and not the imaging parameters. The activation of this mode is questionable for Cine-PC, even if a specific protocol optimization is required to increase SNR and VNR.

Our results showed a complex behavior of phase-offsets depending on *Venc*, scan time, first order moments, gradient amplitude, and slew-rate. As such, velocities measured with lower *Vencs* are more susceptible to errors from intra-voxel dephasing [14]. Knowing the interaction between hemodynamics and hydrodynamics, a modification of any variable generates a derivative change in intracranial volume that leads to a temporal modification of intracranial pressure [25]. These small variabilities might draw a line between low-to-mild, or mild-to-severe alteration in idiopathic intracranial hypertension, Chiari malformation, normal pressure hydrocephalus, or cerebrovascular stenosis [26]. Future clinical works are required to determine the effect of phantom corrections on these pathologies. Future research directions might be an extension of this study to involve other centers to include all major vendors and different gradient strengths. By achieving this, we will have a broader approach to phase-wraparound artifact and the effects on the quantification of brain hemodynamics with PC-MRI.

The present study has few limitations. First, we assumed constant SNR value over the cardiac cycle, hence the effect of the phase dispersion and noise level in the measurement of VNR was neglected, which is a potential limitation. Our aims were to assess the differences between Full-FOV coverage and fold-over using two *Venc* values to encode the CSF and the blood flow dynamics and not to study the overall cerebrovascular system. For this reason, we included the background phase correction for a single artery and not for all of them. We only included a group of 10 healthy controls. Statistical analysis of the fold-over effect using two *Venc* values requires a larger cohort. Finally, the lack of a gold standard technique to measure true flow values on healthy control subjects, to which the Cine-PC values with and without phantom correction could be compared, is another significant limitation to this study.

## 5. Conclusions

We showed that spatiotemporal phase differences vary significantly depending on structure area and *Venc* values. This underlies the needs for protocol-specific calibration measurements and phase-offset correction. The clinically available gradient mode runs with standard derating of gradient performance in order to decrease inaccuracies, and both coverage options still suffer from spatially varying background phase-offset due to eddy-currents and concomitant magnetic field that deteriorate the quantification of cerebral flow in Cine-PC. Compared to full coverage, fold-over oversampling is an alternative to increase spatial resolution and provides comparable flow quantification values. This option can be selected with Cine-PC phase-difference reconstruction when low *Venc* values are prescribed for the assessment of cerebral brain flows.

## Figures and Tables

**Table 1:** Imaging parameters. The table shows the similarities and the differences in terms of image acquisition parameters for both options (Full-FOV and fold-over) using the 2 *Venc* values.

	*Venc* = 600 mm/s		*Venc* = 100 mm/s	
	Full-FOV	Fold-Over	Full-FOV	Fold-Over
**TR/TE [ms]**	10/6.2	10/6.2	14/8.8	15/8.1
**FOV [mm^2^]**	120 × 120	90 × 80	90 × 80	40 × 40
**Acquisition Matrix**	120 × 120	80 × 80	180 × 160	80 × 72
**Acquisition Pixel [mm^2^]**	1 × 1	1 × 1	0.5 × 0.5	0.5 × 0.5
**Reconstruction Matrix**	240 × 240	128 × 128	288 × 288	128 × 128
**Reconstruction Pixel [mm^2^]**	0.5 × 0.5	0.3 × 0.3	0.3 × 0.3	0.3 × 0.3
**Oversampling [mm x mm]**	No	35 × 35	No	70 × 70
**BW/pixel [Hz]**	191	191	145	144
**Acquisition time**	1′ 17”	1′ 27”	1′ 43”	3′ 49”

**Table 2:** Effects of phase-offset phantom correction. Results of the measurements achieved before and after phase-offset correction. These were performed separately on both coverage options and are obtained in the aqueduct (*Venc* = 100 mm/s) and the internal carotid artery (*Venc* = 600 mm/s). The values are displayed by their mean and standard deviation. Bilateral paired statistical t-test was performed to compare the variables separately for each coverage option with a threshold of *p <* 0.05. The bold means *p <* 0.05.

	Aqueduct CSF (*Venc* = 100 mm/s)			ICA Blood (*Venc* = 600 mm/s)		
	Native	Corrected	*p*	Native	Corrected	*p*
**Full-FOV**						
Mean Velocity (mm.s^−1^)	−0.86 ± 6.89	−2.03 ± 5.14	0.59	214 ± 30	223 ± 24	0.05
Peak Velocity (mm.s^−1^)	57.38 ± 20.15	54.42 ± 17.89	0.45	356 ± 45	366 ± 35	0.12
Minimum Velocity (mm.s^−1^)	−33.31 ± 58.24	−62.88 ± 21.36	0.11	135 ± 23	141 ± 18	0.16
Net Flow volume (μL.s^−1^)	−10.98 ± 16	−2.48 ± 12.24	0.07	4200 ± 562	4321 ± 537	0.07
Stroke Volume (mL)	6.36 ± 29.65	8.24 ± 28.97	0.26	1631 ± 837	1684 ± 847	0.05
**Fold-Over**						
Mean Velocity (mm.s^−1^)	−3.13 ± 4.19	−0.74 ± 5.49	**0.009**	208 ± 28	214 ± 22	0.31
Peak Velocity (mm.s^−1^)	33.35 ± 31.81	43.68 ± 15.74	0.22	346 ± 34	355 ± 28	0.17
Minimum Velocity (mm.s^−1^)	−42.78 ± 32.21	−47.96 ± 19.36	0.50	131 ± 23	134 ± 24	0.64
Net Flow volume (μL.s^−1^)	−10.48 ± 14.73	5.85 ± 17.50	**0.02**	4103 ± 500	4196 ± 560	0.31
Stroke Volume (mL)	38.66 ± 25.62	36.94 ± 26.90	0.30	1526 ± 340	1553 ± 340	0.45

**Table 3:** Differences between Full-FOV and fold-over. Results of the measurements achieved to compare Full-FOV and fold-over. Bilateral paired statistical t-test was performed to compare the two coverage options (threshold *p* < 0.05). The bold means *p <* 0.05. These are performed in the aqueduct (*Venc* = 100 mm/s) and the internal carotid artery (*Venc* = 600 mm/s) and are displayed by their mean and standard deviation values. The variables are: velocity-to-noise ratio (VNR), section area of the aqueduct and the internal carotid artery, phase-offset on phantom (mean velocity = V_mean_^offset^, peak or maximum velocity = V_peak_^offset^, minimum velocity = V_min_^offset^), volunteers’ native measures (mean = V_mean_, peak = V_peak_, minimum = V_min_, net flow = Q_Net,_ stroke volume = SVol), volunteers’ measures following phase-offset phantom correction (mean velocity = V_mean_^corr^, peak or maximum velocity = V_peak_^corr^, minimum velocity = V_min_^corr^, net flow = Q_Net_^corr^_,_ stroke volume = SVol^corr^).

	Aqueduct CSF (*Venc* = 100 mm/s)			ICA Blood (*Venc* = 600 mm/s)		
	Full-FOV	Fold-Over	*p*	Full-FOV	Fold-Over	*p*
VNR	1.54 ± 1.99	2.58 ± 1.94	0.25	1.78 ± 0.82	2.49 ± 0.96	0.09
V_mean_^offset^ (mm.s^−1^)	−2.01 ± 3.17	−3.33 ± 3.03	0.36	−8.6 ± 12.4	−5.6 ± 17.1	0.66
V_peak_^offset^ (mm.s^−1^)	3.08 ± 2.65	0.4 ± 2.4	**<0.01**	9.5 ± 12.0	8.8 ± 14.2	0.91
V_min_^Off^ (mm.s^−1^)	−7.03 ± 4.95	−6.75 ± 4.31	0.82	−22.5 ± 13.7	−22.3 ± 20.3	0.84
V_mean_ (mm.s^−1^)	−0.9 ± 6.9	−3.1 ± 4.2	0.39	214 ± 30	209 ± 29	0.66
V_peak_ (mm.s^−1^)	57.4 ± 20.2	33.4 ± 31.8	0.06	357 ± 46	346 ± 34	0.58
V_min_ (mm.s^−1^)	−33.31 ± 58.24	−42.78 ± 32.21	0.64	135 ± 23	131 ± 23	0.47
V_mean_^corr^ (mm.s^−1^)	−2.0 ± 5.1	−0.7 ± 5.5	0.58	223 ± 24	214 ± 22	0.41
V_peak_^corr^ (mm.s^−1^)	54.4 ± 17.9	43.7 ± 15.7	0.17	365 ± 35	355 ± 28	0.47
V_min_^corr^ (mm.s^−1^)	−62.88 ± 21.36	−47.96 ± 19.35	0.08	141 ± 18	134 ± 24	0.15
Q_Net_ (μL.s^−1^)	−10.98 ± 16.00	−10.48 ± 14.73	0.94	4200 ± 562	4104 ± 500	0.69
Q_Net_^corr^ (μL.s^−1^)	−2.48 ± 12.24	5.85 ± 17.50	0.23	4321 ± 538	4196 ± 560	0.62
Section Area (mm^2^)	2.86 ± 0.98	3.47 ± 1.30	**<0.01**	19.78 ± 2.73	19.90 ± 2.86	0.83
StVol (mL)	37.79 ± 28.3	40.56 ± 30.95	0.52	1632 ± 837	1526 ± 340	0.67
SVol^corr^ (mL)	41.58 ± 29.64	40.15 ± 31.18	0.86	1685 ± 848	1554 ± 340	0.59

**Table 4:** Comparison of the two coverage options Full-FOV and fold-over. The Bland–Altman analysis and the Pearson correlation test were performed for each measured variable in the aqueductal CSF and in the internal carotid artery blood flows. M stands for the mean differences between Full-FOV and fold-over. The upper limit U equals *[M +1.96 SD]* while the lower limit L equals *[M − 1.96 SD].* The bias (B) was calculated as M x (variable differences between the two methods)/(variable)], *r* stands for the Pearson correlation coefficient and *p* refers to the significance threshold vale and was set to 0.05. The bold means *p <* 0.05. The variables are: velocity-to-noise ratio (VNR), section area of the aqueduct and the internal carotid artery, phase-offset on phantom (mean velocity = V_mean_^offset^, peak or maximum velocity = V_peak_^offset^, minimum velocity = V_min_^offset^), volunteers’ native measures (mean = V_mean_, peak = V_peak_, minimum = V_min_, net flow = Q_Net,_ stroke volume = SVol), volunteers’ measures following phase-offset phantom correction (mean velocity = V_mean_^corr^, peak or maximum velocity = V_peak_^corr^, minimum velocity = V_min_^corr^, net flow = Q_Net_^corr^_,_ stroke volume = SVol^corr^).

	Fold-Over Versus Full-FOV											
	Aqueduct CSF						Internal Carotid Artery Blood					
	Bland–Altman				Pearson		Bland–Altman				Pearson	
	M	L	U	B	r	*p*	M	L	U	B	r	*p*
V_mean_^offset^ (mm.s^−1^)	1.3	−3.0	5.6	−13.3	0.75	**0.012**	−3.0	−21.5	15.5	0.5	0.84	**0.002**
V_mean_ (mm.s^−1^)	2.3	−15.5	20.0	6.7	−0.29	0.41	5.8	−29.7	41.3	0.2	0.81	**0.004**
V_mean_^corr^ (mm.s^−1^)	−1.3	−11.45	8.88	0.90	0.52	0.12	8.7	−30.4	47.9	0.3	0.63	**0.048**
V_peak_^offset^ (mm.s^−1^)	2.7	−0.4	5.8	1.8	0.81	**0.005**	0.7	−20.2	21.6	−0.9	0.68	**0.030**
V_peak_ (mm.s^−1^)	14.7	−14.2	14.6	4.3	0.52	0.12	10.1	−57.6	77.8	0.3	0.66	**0.039**
V_peak_^corr^ (mm.s^−1^)	10.7	−4.7	26.2	2.4	0.90	**<0.001**	10.4	−63.2	84.0	0.3	0.31	0.39
V_min_^Off^ (mm.s^−1^)	−0.3	−8.0	7.5	0.001	0.64	**0.045**	−0.2	−20.9	20.6	0.1	0.88	**<0.001**
V_min_ (mm.s^−1^)	15.5	−111	142	71	0.15	0.68	3.2	−23.4	29.8	0.1	0.82	**0.003**
V_min_^Corr^ (mm.s^−1^)	−14.9	−61.6	31.8	−3.9	0.32	0.37	7.0	−20.9	34.9	0.4	0.80	**0.005**
Q_Net_ (μL.s^−1^)	−0.5	−26.9	25.9	0.2	0.62	0.06	97	−428	621	2.1	0.85	**0.002**
Q_Net_^corr^ (μL.s^−1^)	−8.3	−45.7	29.0	3.7	0.21	0.55	125	−444	693	3.7	0.85	**0.002**
SVol (mL)	32.3	−30.6	95.2	−3.6	0.90	**<0.001**	106	−1361	1572	−0.6	0.45	0.19
SVol^corr^ (mL)	−28.7	−89.7	32.3	0.9	0.68	**0.030**	−131	−1606	1343	1.0	0.47	0.17
Area (mm^2^)	−0.6	−1.7	0.5	0.1	0.91	**<0.001**	−0.1	−3.61	3.36	0.001	0.80	**0.006**

## Abbreviations

Cine-PC	Cine Phase Contrast
CSF	Cerebrospinal Fluid
*Venc*	Velocity Encoding
VNR	Velocity-to-Noise Ratio
V_mean_	mean flow velocity
V_mean_^corr^	corrected V_mean_
Q_Net_	Net Flow
Q_Net_^corr^	corrected Q_Net_;
SVol	Stroke Volume
SVol^corr^	corrected SVol;
ICA	Internal Carotid Artery;
FOV	Field-Of-View.
